# Frequency-Division Multiplexing for Electrical Impedance Tomography in Biomedical Applications

**DOI:** 10.1155/2007/54798

**Published:** 2007-09-06

**Authors:** Yair Granot, Antoni Ivorra, Boris Rubinsky

**Affiliations:** ^1^School of Computer Science and Engineering, Hebrew University of Jerusalem, 78b Ross Building, Jerusalem 91904, Israel; ^2^Biophysics Graduate Group, University of California, Berkeley, CA 94720-3200, USA; ^3^Department of Mechanical Engineering, University of California, Berkeley, CA 94720-1740, USA

## Abstract

Electrical impedance tomography (EIT) produces an image of the electrical impedance distribution of tissues in the body, using electrodes that are placed on the periphery of the imaged area. These electrodes inject currents and measure voltages and from these data, the impedance can be computed. Traditional EIT systems usually inject current patterns in a serial manner which means that the impedance is computed from data collected at slightly different times. It is usually also a time-consuming process. In this paper, we propose a method for collecting data concurrently from all of the current patterns in biomedical applications of EIT. This is achieved by injecting current through all of the current injecting electrodes simultaneously, and measuring all of the resulting voltages at once. The signals from various current injecting electrodes are separated by injecting different frequencies through each electrode. This is called frequency-division multiplexing (FDM). At the voltage measurement electrodes, the voltage related to each current injecting electrode is isolated by using Fourier decomposition. In biomedical applications, using different frequencies has important implications due to dispersions as the tissue's electrical properties change with frequency. Another significant issue arises when we are recording data in a dynamic environment where the properties change very fast. This method allows simultaneous measurements of all the current patterns, which may be important in applications where the tissue changes occur in the same time scale as the measurement. We discuss the FDM EIT method from the biomedical point of view and show results obtained with a simple experimental system.

## 1. INTRODUCTION

 Electrical impedance tomography (EIT) is an imaging technique that produces an image of the spatial distribution of the electrical impedance of an object from electrical measurements made with an electrode array on its periphery [1–3]. Image reconstruction in EIT is an inverse problem in which the electrical impedance of a domain is determined from the solution of Laplace's equation with the boundary conditions specified by electrode measurements. In a typical procedure, electric current is injected and removed through a pair of electrodes, while the resulting potentials are measured at other electrodes. The solution of Laplace's equation, which best satisfies all of the boundary conditions would reveal the desired impedance distribution. Increasing the number of electrodes and the number of current injection pair combinations can improve the quality of the EIT image [4]. However, when this is done in a serial manner, data collection might be time consuming, which is detrimental when the conductivity distribution is changing dynamically.

In stationary EIT measurements, the data collection time is not a parameter, but in dynamical scenarios, there is a need to address the changing conductivities over time. In some instances, these changes are actually used to perform temporal differential EIT with biological tissue. Comparing the results from two measurements can show the areas where the conductivity has changed over time, but the main theme of this study is not concerned with differential EIT. When one measures an unstable quantity, the measurement should either be short enough so that the quantity is essentially constant throughout the measurement, or alternatively, consider the changes during the data acquisition period. One approach to account for the changes uses a Kalman filter [5] to track them from one voltage measurement to the next. It assumes that during the relatively short time of a single current injection, the conductivity is constant. Although this is not enough to reconstruct the image, the conductivity may already be estimated and it is modeled as a dynamically changing variable. The values for the next voltage measurement are predicted by the Kalman filter according to this model and are refined as more and more measurements are made.

There are several other approaches to deal with dynamically changing situations [6, 7] that acknowledge the fact that the impedance is changing from one measurement to the next. These usually account for this rapid change in the data processing phase and are used in many cases where the change is cyclic, for example, in cardiopulmonary monitoring. We are interested in cases where the change may not be cyclic but rather in a transition from one state to another. We would also like to be able to complete the data collection phase before any noticeable change occurs, so that there will be no need for using special techniques in the data processing phase. Other solutions include the use of extremely short pulses that may obtain up to 1000 frames per second, but these are used mostly in industrial applications [8].

In this paper, we propose a different method of dealing with rapidly changing conductivities, namely, injecting and measuring the effects of all the current injecting electrodes simultaneously and using the principle of superposition in the decomposition and analysis of the output data. Simultaneously injecting currents from multiple electrodes has been suggested in the past as a method of controlling the current distribution within the body for the adaptive current technique [9]. Yet, in the adaptive current technique, it is impossible to distinguish between the effects of the various current sources because of the linearity of the equation. If the currents from the different sources are each injected at a unique frequency, a Fourier decomposition of the resulting measured voltages would match the injecting currents, and the effects of each injecting current electrode could be isolated. This way, rather than performing numerous measurements with different current injecting electrodes and using linear superposition to analyze the data, like in conventional EIT, we can perform one single measurement in which each injecting electrode uses a different frequency, and we use the superposition principle with Fourier decomposition for the output data. This is called frequency-division multiplexing (FDM) EIT. A similar concept was also proposed for industrial applications of EIT [10]. That study uses neural networks, and its focus is data collection speed in industrial applications. Our research was originally motivated by the need to perform simultaneous measurements to capture a specific instant in the dynamically changing electrical properties of tissue and was developed for monitoring minimally invasive surgery such as cryosurgery and electroporation [11–13]. In this paper, we analyze the situation using the finite element method (FEM) and address the important implications of imaging biological tissues. Probably, the most important issue to consider from the FDM point of view in biomedical applications is the frequency dispersion. We show how to account for the dispersions and analyze the errors that may arise due to them.

Living tissue is a heterogeneous material characterized by the presence of the cell membrane, which has a high capacitance and a low but complex conductance, and by the electrolytic conductive intra- and extracellular domains. Various types of tissue have different frequency dependent electrical properties. Nevertheless, in all tissues, the conductivity increases and the relative permittivity decreases from their low frequency values to the high frequency limits, not gradually but rather in three major steps, termed the *alpha*, *beta*, and *gamma* dispersions. These dispersions occur in the frequency ranges of below 1 kHz, 100 kHz–1 MHz and above 100 MHz, respectively [14]. Several reviews on this topic, as well as compilations of data, have been published [15–18].

**Figure 1:**
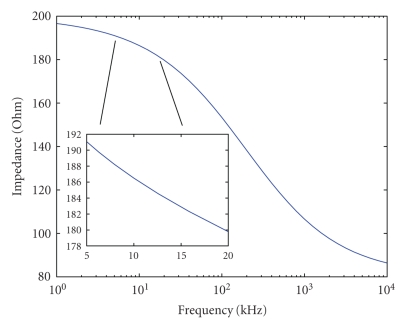
Typical example of a beta dispersion in a tissue. A specific band between 5 kHz and 20 kHz is shown in more detail to demonstrate the nearly linear relation.

When working with tissues using multiple frequencies, one must consider the implications of the different dispersions. In Figure 1, we demonstrate a typical beta dispersion. The change in the tissue impedance is given as a function of frequency. We highlight a small section between 5 kHz and 20 kHz where our actual FDM EIT measurements were performed. Notice the considerable change in impedance between 100 kHz and 1 MHz. Since different electrodes use different frequencies, the associated impedance that is measured will vary as well. When the electrical properties of the different tissues that are measured are known or can be approximated, we are able to overcome this problem. Two possible solutions are either to choose a frequency band where the change is very small or to model this change and compensate for it when the data is processed. For example, in the frequency band between 5 kHz and 20 kHz, shown in Figure 1, the changes in conductivity are relatively small, so we can treat the conductivity in this region as constant. However, since we do have a realistic model for even that small change as a function of frequency, we can compensate for that function and reduce the errors that result from dispersion. This is similar to gauging the system for a specific tissue response. In the results section, we present an analysis of the errors that arise if these changes are not accounted for.

In the past, multiple frequency EIT has been used mostly to differentiate between tissue types. EIT with two different frequencies can be used to create a differential EIT image and detect types of tissue according to their response to the frequency change. In most of the previous multifrequency studies, currents are injected in a serial form from different pairs of electrodes [19, 20]. Simultaneous injections of multiple frequencies for differential imaging were reported before [21], but all of the frequencies were injected through the same electrode. The FDM EIT approach is different. Here, we propose injecting, in each current electrode, a unique frequency that shall identify this electrode. All frequencies are chosen from a narrow frequency band in which the tissue response will be similar. Alternatively, the change can be modeled as explained above, to compensate for known differences. Choosing frequencies that are far enough apart, but still within such a band, we can treat the imaged impedance as independent of frequency. Thus, it is possible to inject multiple currents simultaneously with different frequencies from different electrodes and use Fourier decomposition to independently analyze the voltages related to each injected current. In fact, we use the method of separating the output signal according to frequency as previous authors [21] but give each signal a geometrical meaning, that is, the particular electrode from which it was injected rather than a physiological meaning. In this report, we describe the concept of FDM EIT for biomedical applications, treat the question of choosing suitable frequencies and analyze the errors that result from incorrect assumptions. Finally, we show actual results from a real FDM system, demonstrating that simultaneously injecting several frequencies does not impair the reconstruction process.

## 2. MATERIALS AND METHODS

**Figure 2:**
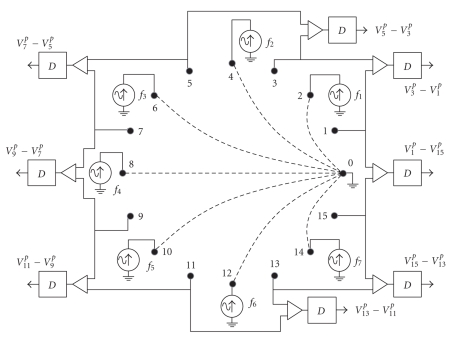
Implementation of a possible FDM EIT system with 16 electrodes. AC currents at different frequencies are injected simultaneously into the sample and are collected by a single electrode (0). Differential voltage at electrode pairs are amplified and processed by demodulators (D).

Figure 2 illustrates the concept of the FDM EIT technique. The figure shows an ideal implementation of a possible FDM EIT system with 16 electrodes. As an example, even-numbered electrodes are used to inject currents into the sample whereas odd-numbered electrodes are employed to measure the resulting voltage differences. Using only half of the available electrodes for each task affects the reconstruction quality, but this is not specific for FDM, and the consequences are identical to traditional systems that apply two sets of electrodes [22]. The currents are simultaneously injected from the current electrodes to a single sink electrode (electrode number 0). Each AC current source has a specific frequency and the voltage differences are demodulated for these frequencies. That is, demodulators (referred to as “D” in Figure 2) have multiple outputs: one for each injected frequency. Therefore, the contribution from each current source at each electrode pair can be isolated. Time is only consumed for the demodulation process but not for multiplexing. As it will be explained later, demodulation time will depend on the spectral separation between the injected currents.

Using several currents simultaneously limits the maximal current levels that may be used due to safety measures. However, this limitation is identical in all of the systems that are using multiple currents, whether these are used for spectroscopy [21], adaptive current technique [9] or other applications [23]. In some biomedical applications [24], the limiting factor may not necessarily be the signal to noise ratio, and then, using the maximal allowed current is not the top priority. When considering the same currents, the treatment of errors in FDM EIT is similar to that of other EIT systems [25].

### 2.1. Hardware

**Figure 3:**
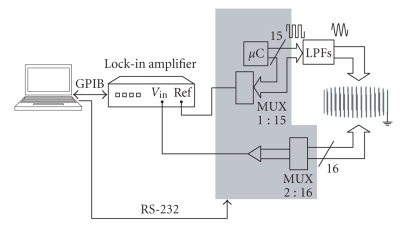
Implemented system for demonstrating the feasibility of the FDM EIT technique. The electrode setup and the liquid container are represented on the right.

In order to demonstrate the feasibility of the FDM EIT technique and to assess possible implementation methods, we have built a simplified version of the above-stated scheme with a single single-channel demodulator (Figure 3). Multiplexing for voltage pairs is required and that makes its time consumption performance equivalent to that of previous EIT systems. However, since simultaneous current injection at different frequencies is performed, this low-cost platform is valid to demonstrate the FDM EIT concept.

The system is composed of 32 stainless steel electrodes around a circular liquid container (diameter = 65 mm), where 15 electrodes are used as current sources, one for the current sink, and 16 for voltage measurements. Each electrode injects a current at a slightly different frequency. These frequencies are all in a frequency band for which the conductance of saline solutions is constant between 5 kHz and 20 kHz. All of the currents had an amplitude of 80 μA.

The injected AC currents are obtained from square signals generated by a set of low cost micro-controllers (μC) that are filtered by second-order low-pass filters (LPFs) with a quality factor (Q) of 4 and centered at the frequency of interest. A different filter was used for each current source and tuned appropriately for the frequency of that current source. A more expensive but probably more convenient solution for future designs could be based on direct digital synthesis techniques.

Voltage signals are simply converted into current signals by means of 100 kΩ output resistors. This creates some errors due to the impedance of the sample and the electrodes. However, in the experiments presented here with saline solutions, such an error is much lower than 1% and does not create significant problems. In other cases, it could be convenient to make use of current sources with much higher output impedances, for instance, based on Howland configurations [26].

The demodulation process is performed by a commercial lock-in amplifier (Model 7280 BFP, Signal Recovery, Oak Ridge, TN, USA). A 1:15 multiplexer (MUX) is used to select one of the current generation signals as the reference signal (Ref) for the demodulation. The measurements are read by a PC that is also responsible for controlling the multiplexer's signals.

**Figure 4:**
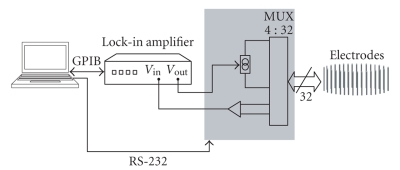
Implemented traditional EIT system for comparison.

A classical implementation of a serial single frequency EIT system was also implemented for comparison (Figure 4). In this case, however, the current source is based on a modified Howland circuit [26]. The distribution of current and voltage electrodes is the same as in the case of the FDM EIT system. Thus, the results provided by this EIT system should be equal to those obtained by the FDM EIT system. Because of that, we will refer to this system as the “emulation” system.

### 2.2. Signal analysis

Every current in the FDM system is assigned a unique frequency. It is usually associated with a specific electrode, but in general, several frequencies could be injected through the same electrode in parallel. In the following derivation, we assume that only a single current is injected through each electrode and thus there is a one-to-one mapping between the frequency and the electrode.

The current *I_p_*injected to electrode *i_p_*, at frequency *f_p_*, is

(1) $$I_{p}=A_{p}\exp(j(2\pi f_{p}t+\phi_{p})),$$

where *A_p_* is the signal amplitude, $\phi_{p}$ is the signal phase, and *t* is the time. The frequency in our example is $f_{p}=4+p$ kHz, the electrode number is $i_{p}=2p$, and p=1,2,…,15.

The odd-numbered electrodes measure the voltage in all of the frequencies simultaneously. In order to separate the signals, care must be taken that the bandwidth of each signal does not exceed the difference between two adjacent frequencies. In our example, we take 15 frequencies between 5 kHz and 19 kHz with a frequency separation of 1 kHz. If the signal's frequency band extends beyond this gap, signals will mix. This is known as intersymbol interference (ISI). The signal bandwidth, ΔF, is inversely proportional to the signal's duration, ΔT, so we require

(2) $$\Delta T\geq \frac{1}{\Delta F}=1\;\text{msec}\text{.}$$

This means that the signal we use to make the measurements (the injected current) cannot be shorter than one millisecond. Longer signals or demodulation time will lower the effects of ISI. When sampling the voltages, the exact duration of the signal may be modified slightly so that all of the injected signals complete an integral number of cycles.

Consider the case of a single current injection. We inject current *I_p_* to a current injection electrode *i_p_* at frequency *f_p_*. We then measure the voltage $V_{l}^{p}$ on a voltage measurement electrode *l*:

(3) $$V_{l}^{p}=B_{p,l}\exp(j(2\pi f_{p}t+\Phi_{p,l})).$$

We define *B_p,l_* and $\Phi_{p,l}$ to be the amplitude and phase, respectively, at the voltage measurement electrode *l* that results from this single injection. Now, we inject several currents simultaneously through all the current injection electrodes $i_{p}=2,4,6,\ldots ,30$. The integrated voltage *V_l_* measured at the voltage measurement electrode *l* is the superposition of the voltages in all of the frequencies. The integrated voltage is then

(4) $$V_{l}=\sum_{p=1}^{15}V_{l}^{p}=\sum_{p=1}^{15}B_{p,l}\text{exp}(j(2\pi f_{p}t+\Phi_{p,l})),$$

where l=1,3,…,31.

The signals can be separated by using a Fourier transform. For each *V_l_*, the 15 values of *B_p,l_* and $\Phi_{p,l}$ are extracted from the discrete Fourier transform of the sampled voltage [21]. Alternatively, the voltage can be measured directly for each frequency with the appropriate test equipment as shown in Figure 3.

Each voltage component, $V_{l}^{p}$, is used independently in the reconstruction algorithm in a similar manner to measurements that were acquired serially. In this case, we have 240 such measurements coming from 15 frequencies and 16 voltage measurement electrodes. However, not all these measurements are independent. Since we are measuring the voltages with reference to one another (Figure 2), there are only 15 independent voltage measurements at each frequency. Hence 225 results are used for the reconstruction process.

## 3. RESULTS AND DISCUSSION

The reconstruction was implemented using a Matlab program that is based on the EIDORS 2D package [27]. We have used the absolute value of the voltage for our examples but it is possible to process complex EIT results, where the phase data, $\Phi_{p,l}$, is used as well. We have compared three cases: simulation, emulation, and real measurements. In the simulation, we have analyzed the situation theoretically, creating a mesh with an inhomogeneity and solving the forward problem to compute the voltages of the voltage measurement electrodes. Then using these values as input, we solved the inverse problem to come up with an image of the inhomogeneity. In the emulation, a traditional EIT system, as shown in Figure 4, was used. Current was injected sequentially into the current injection electrodes, and measurements were recorded with the voltage measurement electrodes. A single frequency was used for the electrodes at each test. The most important results are these of the FDM system itself. These results show that the FDM EIT method can be used to reconstruct an image in a realistic scenario.

The real measurements included 15 current sources, as described above, at frequencies between 5 kHz and 20 kHz which were injected simultaneously from all the current injecting electrodes to the current sink. At the same time, measurements were made using the voltage measurement electrodes.

**Figure 5:**
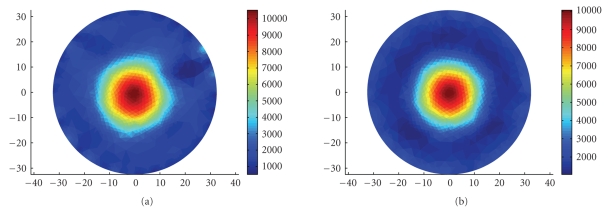
FDM EIT reconstruction of a glass disk (20 mm in diameter) is in the center of a circular saline tank. (a) FDM measurements. (b) Emulation results. The scale shows the impedance in Ω cm.

We have tested the system with a circular saline tank 0.09% NaCl, 65 mm in diameter, in which we placed a circular glass object with high impedance, 20 mm in diameter. Figure 5 depicts the results of the reconstruction process using the emulation data and the real measurements data. It can be seen that in both cases, the reconstructed image clearly shows a circular high impedance object in the center of the tank.

**Figure 6:**
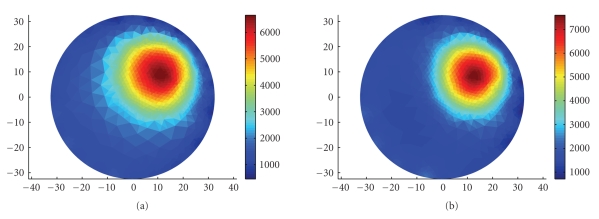
FDM EIT reconstruction of a glass disk in a circular saline tank. (a) FDM measurements. (b) Emulation results. The scale shows the impedance in Ω cm.

The same test was repeated with the object placed close to the current sink electrode. Similar results were obtained. Figure 6 shows the images that were reconstructed from the emulation data and the real measurements. Again, we see a clear circular object near the current sink electrode. The image quality demonstrates the ability of FDM EIT to separate the various frequencies and treat them independently.

Using a single current sink means that the current density near the sink electrode is higher than in other parts of the tank. We, therefore, tested the accuracy of imaging an object that is placed near this electrode, and compared it to other locations. We ran 32 simulations of a small circular object with lower impedance, which at each run was placed close to one of the 32 electrodes. The electrodes are uniformly spaced around the disk at distances of 360/32=11.25 degrees.

**Figure 7:**
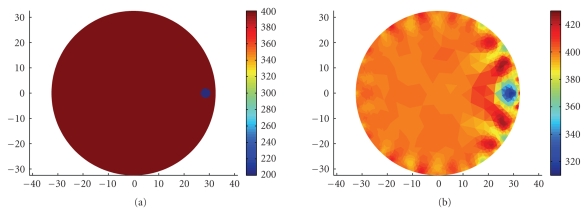
Example of an inhomogeneity near any of the electrodes and its reconstruction. (a) The simulated inhomogeneity. (b) The reconstructed map. The scale shows the impedance in Ω cm.

Figure 7 depicts an example of a synthetic inhomogeneity that was positioned next to one of the electrodes and its reconstructed image. For each inhomogeneity, the injected currents were simulated and the resulting voltage measurements were computed. With the computed voltages as an input, the reconstruction process was run to obtain the estimated impedance inside the tank. These values were then compared to the true impedance with which we started and thus the error was obtained. We repeated the same process with the trigonometric current pattern [28] implemented using half of the electrodes for current injection and half for voltage measurement. In this current pattern, the electrodes inject a combination of sine and cosine functions at the same frequency and the process is repeated 16 times: each time changing the injected current in the electrodes. It is worthwhile noting that the trigonometric current pattern we have used does not have a particularly high current density near any of the current electrodes. Comparing the errors of the two patterns will allow us to determine whether the FDM current pattern has any particular regions of better or worse accuracy.

**Figure 8:**
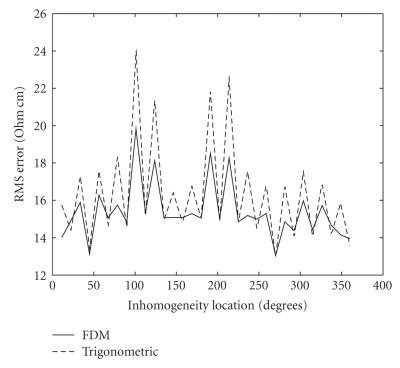
Error of the reconstruction for the trigonometric current pattern and the FDM pattern as a function of the inhomogeneity location.

The performances of the FDM and trigonometric current patterns, according to Figure 8, are quite similar. Both methods show comparable errors for different angles of the object's location. The odd-numbered electrodes (11.25°, 33.75°, etc.) are the voltage measurement ones and the error in front of them is somewhat larger than in front of the current injecting ones. This is probably due to the higher current density in this area, which is why it affects both current patterns. The error does not change considerably when the inhomogeneity is moved around the disk's perimeter but with FDM, it is slightly smaller than with the trigonometric pattern in front of the current sink. This can be seen at the edges of the figure since the sink is at 360°, which is also 0°. The smaller errors in front of the sink electrodes may be explained by the fact that the current density is higher at this location in the FDM pattern but not in the trigonometric current pattern.

Since all measurements are corrupted by noise, it is important to test the noise sensitivity of the method. We have tested the FDM current pattern with additive white gaussian noise tainting the voltage measurements and compared the performance to that of the trigonometric current pattern. The test was performed with simulated inhomogeneities at different locations around the disk. The root mean square (RMS) error was computed and averaged for the entire set of tests at a given noise level.

**Figure 9:**
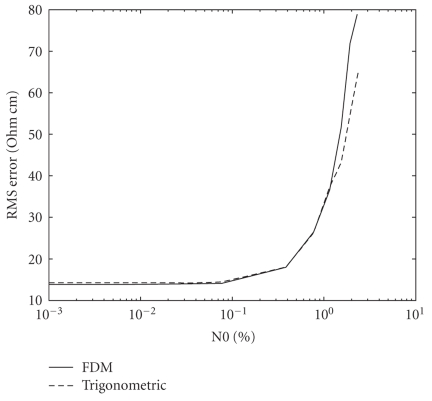
Performance of the FDM and trigonometric current patterns for different noise levels tainting the voltage measurements.

The results of this test are depicted in Figure 9 that shows the RMS error as a function of the noise level, N0. N0 is the standard deviation of the gaussian noise, given in terms of percent of the RMS of the voltage measurements. The tolerance of both current patterns to noise is identical, and both perform well up to noise levels of about 0.5%. At higher noise levels, no clear image is reconstructed, and the difference between the methods is insignificant.

In many biomedical applications, SNR is a limiting factor due to safety concerns regarding the maximal injected current. In the frequency band between 1 kHz and 100 kHz, the current limit increases linearly with the frequency, so a current injected at 20 kHz, for example, can be twice as large as a current at 10 kHz, and still be within the safety limits [23]. In our default configuration, all of the currents are injected at the same amplitude but due to the smaller effect of the currents at higher frequencies, this amplitude will be higher than the amplitude of a system using only lower frequencies, and lower than that of a high frequency system. Since we are using pure sine waves, the RMS current will simply be 0.707 times the amplitude. The SNR depends of course also on the noise level, which is associated with the specific implementation of the measurement device, but should not be different than those of other multiple current systems.

One of our major results is the effect of frequency dispersion on the FDM system. Our assumption is that the measurements are performed in a limited bandwidth in which the tissue conductivity is constant, or almost constant for every frequency. But it is important to check the consequences of using the FDM method when this assumption is not completely fulfilled. To do that, we have tested a scenario in which the conductivity of the tissue does change with frequency. Assuming that the conductivity changes in a linear fashion, we examine the difference in the reconstructed image when the effective conductivity distribution is slightly different for each electrode. In this case, using frequencies between 5 kHz and 19 kHz, we look, for example, at a change of 1% per kHz. So, the first current injected electrode at 5 kHz refers to the original conductivity, 1σ, for the second one at 6 kHz, the effective conductivity is 1.01σ, for the third at 7 kHz, it is 1.02σ, and so forth.

**Figure 10:**
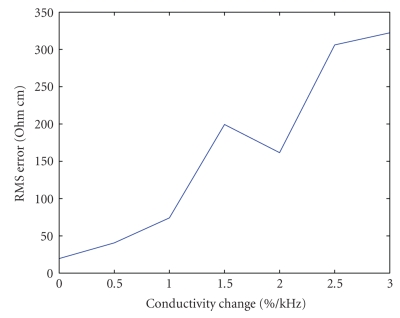
Error in reconstruction caused by large changes in conductivity as a function of frequency

The simulation result depicted in Figure 10 is of a circular object with a radius of 10 mm at the center of a disk with a radius of 32.5 mm. The figure shows the RMS error as a function of the conductivity's dependence on frequency. The graph starts with 0% per kHz (no changes at all) and displays the results for changes of up to 3% per kHz. The relative errors are small and the image is reconstructed in a comprehensible manner up to changes of 1% per kHz. This is a large dispersion that is not common in most tissues. In this example, this dispersion demonstrates a successful reconstruction with a difference in conductivity of up to 15% across the frequency band. This demonstrates that it is possible to use much wider frequency bands and still obtain a good image with FDM EIT.

## 4. CONCLUSIONS

A method for simultaneous collection of EIT measurements was proposed and analyzed. A prototype was built and tested to show that this method can give results that are comparable with traditional data collection methods that serially inject current through electrode pairs. Our tests confirm that it is possible to inject several currents concurrently, even within a narrow frequency band, and still separate them and treat them independently. This method is relatively robust to noise in the voltage measurements and to dispersion effect in most tissues. The concept described here for electrical impedance tomography could be also implemented in other imaging techniques dealing with electromagnetic fields, such as magnetic impedance tomography.
